# Recent advances in urban system science: Models and data

**DOI:** 10.1371/journal.pone.0272863

**Published:** 2022-08-17

**Authors:** Elsa Arcaute, José J. Ramasco

**Affiliations:** 1 Centre for Advanced Spatial Analysis, University College London, London, United Kingdom; 2 Instituto de Física Interdisciplinar y Sistemas Complejos IFISC (CSIC-UIB), Campus UIB, Palma de Mallorca, Spain; Potsdam-Institut fur Klimafolgenforschung eV, GERMANY

## Abstract

Cities are characterized by the presence of a dense population with a high potential for interactions between individuals of diverse backgrounds. They appear in parallel to the Neolithic revolution a few millennia ago. The advantages brought in terms of agglomeration for economy, innovation, social and cultural advancements have kept them as a major landmark in recent human history. There are many different aspects to study in urban systems from a scientific point of view, one can concentrate in demography and population evolution, mobility, economic output, land use and urban planning, home accessibility and real estate market, energy and water consumption, waste processing, health, education, integration of minorities, just to name a few. In the last decade, the introduction of communication and information technologies have enormously facilitated the collection of datasets on these and other questions, making possible a more quantitative approach to city science. All these topics have been addressed in many works in the literature, and we do not intend to offer here a systematic review. Instead, we will only provide a brief taste of some of these above-mentioned aspects, which could serve as an introduction to the collection ‘Cities as Complex Systems’. Such a non-systematic view will lead us to leave outside many relevant papers, and for this we must apologise.

## Introduction

Cities, and urban systems in general, present generic patterns, such as Zipf’s law [1], despite being the result of a diverse set of processes and constraints. In this section, we look at some of the old and more recent attempts to encapsulate these within a mathematical framework [2], although we will not be able to do justice to all the efforts in the last decades towards an integrated science of urban systems [3]. One example pertains to the agglomeration effects that have been observed worldwide for over a century [4]. This has led researchers to investigate further the effects of city size on urban indicators [5–7]. The topic of urban scaling laws sprouting from this initial idea, has generated a lot of activity, and through the scrutinization of the limitations of the method, see for e.g. [8], new paradigms and models have emerged [9]. The scaling framework on the other hand, does not consider the heterogeneities within cities, nor the mechanisms giving rise to the observed urban metrics. And although many of these are still an open problem, these processes take place within the spatial fabric of the city, and their physical embeddedness cannot be disentangled from their effect. In this sense, many of the spatial correlations of the different processes taking place in cities, are tightly related to the spatial distribution of functions and transport, which are both closely linked to the morphology of cities. Such an interdependency is yet to be understood. Advancing this field necessitates a quantification of the *form* of the city, and although all cities look different, they all reveal fractal patterns [10, 11]. These patterns play a role in modulating the intensity of the interactions between functions. Sometimes, the modulating distance is not necessarily physical, and corresponds to a proxy of higher probability of interaction due to similarity or complementarity between the components. And it is the different intensities of interactions within systems and across scales that give rise to another generic pattern observed in most complex systems: a hierarchical organisation [12].

## Urban scaling laws: What is missing?

Cities can be regarded as the solution to the problem of sustaining the many necessities of human beings. By agglomerating in an area, individuals have been able to share resources and facilitate exchanges allowing for better productivity. Over time, different processes, such as trade, skill matching and specialisation, and the evolution of transport to mention a few, have come together shaping the spatial distributions of land uses in cities. Different models attempting to explain the observed patterns have been proposed since the 19th century, such as von Thünen’s model of concentric rings of land uses as detailed in his treatise of *The Isolated State* in 1826, Christaller’s Central Place Theory [13] aiming at explaining a hierarchical order in the distribution of settlement sizes and their functions, and Lösch’s location theory [14] where he emphasized that transport cannot be disentangled from the observed agglomerations. Overall, for more than half a century, there have been many proposals looking at cities from the perspective of flows [15], and of complexity science through Berry’s proposal to consider cities as “systems of systems” [16]. It is beyond the scope of this paper to provide a comprehensive review of the different theoretical and modelling frameworks to cities, it is nevertheless fundamental to mention two of the pioneers and driving forces behind the development of a science of cities within the framework of complexity science: Denise Pumain [17, 18] and Michael Batty [19, 20].

Although the quest to model cities started more than a century ago, capturing all the processes and their interdependencies continues to be a challenge. Emergent patterns, such as agglomeration economies, have been identified since a century ago [4], and continue to be investigated [21, 22]. This is an active topic within the field of New Economic Geography [23, 24], where sophisticated models continue to be developed to capture the different intervening externalities and interactions, giving rise to the observed spatial unevenness. These ideas were extended beyond the realm of economics, and simplified through the following relationship:

$$\begin{array}{c} Y∼P^{\beta }, \end{array}$$ (1)

where the output *Y* corresponds to an urban indicator, *P* to the population of a city, and the exponent *β* indicates if there are effects due to concentration of people, i.e. if *β* > 1 the output *Y* is more than proportional to the amount of people *P* in the city [5]. A cautionary note on the observed output was brought forward by Denise Pumain [6, 25]. Within the industrial sector, the value of the exponent will not always be larger than 1, but it will depend on the level of maturity of the sector. For example, at the stage when an industry is producing an innovation, the activity is mostly concentrated in big cities, and therefore the expected agglomeration effects with *β* > 1 will be observed. After this initial adoption period, the exponent will shift to *β* ∼ 1, indicating that productivity is no longer concentrated in big cities, but has diffused to smaller ones where production might be cheaper due to lower rents and wages. Similar findings related to the value of the exponent with the phase of economic growth can be found in [26].

Scaling laws in urban systems attracted a lot of interest in many different areas [27–31], and some contradictions started to emerge, touching upon important questions related to the role of city size: are larger cities greener [32–34]? Are they more congested [35, 36]? And are larger cities more unequal [37]? In addition, limitations of the method were also identified: does the result depend on the definition of the system of cities [8]? Or are these contradictions the result of a poor statistical modelling framework [9, 38–40]? Different models have been constructed in an effort to explain the emergence of scaling laws, see for example [41, 42]. In [43, 44] for example, the authors derive the different exponents from the expected interactions and fractal structure of the space through two different approaches. For a more holistic review of the proposed models see [45]. Another important topic which is still subject of research, relates to the reconciliation between transversal and longitudinal scaling to better understand the evolution of cities, see [46–48]. Transversal refers here to scaling as the one defined in Eq (1), while longitudinal includes time as a variable (evolution of scaling laws). It is also expected that such laws would hold for past and contemporary settlements, as has been found in [49]. Overall, urban scaling laws need to include the interaction between cities [50], and their correlations [51], which has not been the case previously. It is well-known within the study of out of equilibrium systems that power laws might arise from single processes leading to a homogeneous relationship such as the simplistic equation described above [52]. Nevertheless, when many correlated processes intervene, homogeneity is lost. In this sense, when thinking about a system of cities, cities are not independent and isolated systems. The correlation between the different processes inside cities needs to be untangled [53], in addition to considering their co-evolution [25].

In a paper in this collection [50], the author considered proximity and interactions between cities, and found that the value of the exponent changes under such considerations. In [51], the authors looked at 96 countries and found that the effect of urban scaling of GDP is correlated to the population distribution. On the other hand, the authors in [54] showed that the observed Zipf’s law emerges from the autocorrelation of the distribution of cities. These latest works emphasize the increasing importance of integrating interactions between cities and correlations into any theory or modelling of cities. The importance of transport as an enabler of interactions, shaping the distribution of functions and evolution of the form of the city, has been pointed out for over a century ago. Nowadays, many of the interactions are channelled over the internet, making the physical distance less relevant for some of the processes. Understanding the extent of the impact of new technologies, in particular of those as widespread as this one, is essential and it is still under research. The authors in [55] contribute to this discussion, by analysing the impact of information and communication technologies (ICT) on the agglomeration benefits, and by pointing out its effect on the distribution of city size.

## Morphology and inequality

The previous section mentioned the importance of transport in facilitating and enabling interactions and different types of flows between the system’s components. In the following sections, we will look in more detail at some of the models of flows. What is important to note at this stage, is that the spatial patterns observed in cities are the outcome of years of restructuring, where positive feedbacks might have reinforced certain paths. Such a reinforcement is the outcome of bottom-up self-organising processes and top-down interventions. These, however, do not necessarily lead to more resilient, nor optimal cities. The *form* of a city can hence be seen as the outcome of all the above-mentioned processes, where no unique solution can be defined. The quest of identifying the interplay between *form* and *function* has been ongoing for many decades now: does form follow function, as Sullivan [56] categorically expressed as a *law* for the design of buildings in 1896? Or is there a feedback process for function to adapt to form and vice-versa? Their co-evolution is a complex mechanism involving slow and fast dynamics. In the seminal work of Christopher Alexander “The city is not a tree” [57], form and function were decomposed into different elements of the city, with a given probability of interacting according to their spatial distribution and the existing flows. Since the elements can be considered land uses, people, etc, greater mixing and diversity of interactions translates into the possibility of experiencing diverse land uses, and diversity of encounters among individuals of different areas. If the city takes a “tree-like” interaction-structure, this means that mixing among elements is minimised, leading to segregation of land uses, and/or people. Such a perspective cannot be detached from transport, which plays a fundamental role in shaping the possible interactions as it co-evolves with land use [58]. Michael Batty discusses in [20], how the debate deepens further when considering the digital world and new technologies. For the interested reader, further reflections on Alexander’s legacy after 50 years of his seminal work can be found in [59].

Let us now look at quantifying *form*, which can be done in a variety of ways. For example, urban morphology can be thought in terms of the shape of its built components, such as the plots and buildings constituting cities [60–62]. This cannot be disentangled from the *age* of the buildings, since the introduction of new buildings into the city is very much dependent on the probability of buildings being demolished and plots being repurposed [63, 64]. As cities evolve, some of the reinforced patterns correspond many times to negative characteristics, such as segregation and poverty [65]. Understanding the effect of the physical form on segregation is an urgent and important problem to address. On the other hand, other reinforcement processes have left a physical imprint corresponding to the street network. And although these are the outcome of different intervening constraints around the world: socio-political, historical, and geographical; the emergent pattern can universally be recognised as a fractal, as initially proposed by Michael Batty [10] and Frank Frankhauser [11]. Furthermore, through the advancement of network science [66, 67], street networks have been analysed through the application and development of different centrality measures [68–75]. In parallel, a whole discipline emerged from the effort of connecting the form of cities through network-like approaches to its function: *space syntax* [76]. In [77], the author discusses the potential mechanisms giving rise to the observed emergent morphologies, from the growth of the road network, to the shape of plots and the distribution of buildings.

With the advent of sophisticated computational methods allowing for the collection, manipulation, and classification of big datasets, a characterisation of cities at a large scale has been possible [78], in particular making use of methods from machine learning [79–81]. These methods have been refined through the combination of different datasets, including remote sensing data, such as LiDAR [82–84].

As paths got reinforced and cities evolved, the process took place in a non-uniform way, leading to different growth rates in the system. This produced a multifractal organisation of the street network [85, 86], and of urban systems in general [87]. The self-organised processes, together with top-down interventions are responsible of the observed morphologies of cities. London for example, evolved from a multifractal to a mono-fractal, following the introduction of a greenbelt around the city constraining its growth [85]. In more extreme cases, interventions such as the one undertaken by Hausmann in Paris in the late 19th century, have seen large parts of the system destroyed to reconstruct a new order.

The morphology of a city constrains the spatial distribution of functions. Multifractal methods can be used to understand both, the spatial disparities, and the skewness of the distribution [88, 89] leading to inequality. On the other hand, there are proposals on reducing inequality through the redistribution of flows [90]. The topics of inequality and segregation could have their own collection in the journal, and it is not our intention to propose a review of the topics here. It is important nevertheless, to mention their embeddedness within many different layers of the city, including mobility patterns [91, 92]. This allows us to further motivate the role of flows within urban systems as an essential component. For example, these play a central role in the characterisation of spaces [93, 94], and in defining their importance within the system.

## Hierarchical organisation

The previous section revealed that the morphology observed in cities is a consequence of the reinforcement of the connectivity between places occurring in a non-homogeneous way. The heterogeneous interactions take place at different scales. For example, at a very granular level, denser parts of the city have a higher probability of encounters, and these could be identified as neighbourhoods. At the next level, cities can be represented in terms of their neighbourhoods as nodes, which are many times defined in terms of administrative boundaries, such as boroughs or census tracts. Neighbourhoods also present different degrees of interaction between them, which can be characterised in terms of commuting flows [95], or any other type of interaction proxy [96]. This generates a nested structure which encodes a hierarchical structure where different parts of the system are more connected than others, generating feedback loops across scales. Such a structure is not confined to cities; interactions can be defined between cities, regions, countries, or between the different scales [97]. Furthermore, hierarchical organisation is a commonly observed pattern in complex systems [12, 98].

At this point, it is important to recall that many of the observed processes are the outcome of interactions which have been modulated by their spatial distributions and the speed of transportation [99]. Hence, the co-evolution of different systems within cities is coupled to technological advancements. And although many of the interactions take place in the cyberspace, mobility in cities also shows a strong hierarchical organisation, see for instance [100–102]. Such an organisation mimics Christaller’s idea in the central place theory for the hierarchy between cities, with some areas being of higher category than others and attracting or emitting most of the trips (hotspots) [103, 104]. This is a mesoscopic level of description of the city’s organisation, since it requires the analysis of aggregated mobility and of the structure of the hotspots and their levels in space. However, as was shown in [100], the fact that a city is widespread or more compact in terms of its centres of activity can be connected to indicators of the quality of life such as the transport modes used to travel to work, the levels of pollution and public health records.

## Urban mobility

The role of mobility in cities is to interconnect the different zones constituting an urban area. There are plenty of reasons why people residing at a certain neighbourhood need to travel like, for instance, to work, to find goods and services, for leisure purposes, etc. As shown by the last results discussed on the hierarchical organisation of cities, both morphological aspects and mobility are strongly entangled. Nevertheless, these two questions have been addressed in the literature as two separate issues. The next important quest is to bring these two fields together. In the meantime, let us look at the work done on mobility, in particular at the data needed to characterise it and at the models proposed to explain it at different scales.

Traditionally, census surveys collect the present residence location of citizens and, in many countries they include a question on the place of residence in the previous census. Since the period between surveys is around 10 years, this information provided a basis for the very first analyses on migration flows [105]. Much later, already in the 2000s for the US for example, the surveys incorporated the question of county of work. In this way, it was possible to outline the commuting mobility flows at country scale. In terms of surveys, there has also been a tradition of performing local travel surveys in several cities. The final goal is to improve the management of the public transport system, but since the questions asked are more specific these are very valuable information sources. Unfortunately, in most of the cases transport surveys are not standardized across cities and they have an eminently local character.

For digital data sources, one of the first works was related to the “where is George?” experiment [106] in which individuals introduced in a web the code of the bank notes across the US. Following the notes through their locations, it was possible to gain insights on the potential travels. However, this could only provide a type of proxy of mobility. Mobile phone records introduced a much more direct way to measure it. The first results focused on land use in different areas of the cities [107–110], while a later work by M. González et al. analysed individual mobility patterns [111]. After that, there has been plenty of work with this type of data, including the analysis of the social network in space, mobility in cities [112], etc (see, for instance, [113] for a review). Other digital sources of data include online social networks as Twitter or Facebook.

### Models of mobility

Theoretical models must adjust to the scales that they intend to reproduce. Mobility can be seen as a personal phenomenon in which individuals or agents crawl across the city, or as an aggregated entity connecting city zones with flows of people travelling between them. The approaches used to address these two scales are based on different levels of knowledge on the system and input data details. While individual-based models attempt to reproduce trajectories, something that requires rules on people decision-making and data on their trip demands, aggregated models need only parameters accounting for the city-zones properties as sources or sinks for travels.

### Individual-based models

The original individual models were based on concepts developed for random walks [114] and Lévy flights (see for instance [115] for a recent review). The main variable is the position *X*_*i*_(*t*) of every agent *i* at time *t* and the metrics are built out of it as, for instance, the mean square displacement per agent or the mean (median) radius of gyration [111]. The basic versions of these models lead to diffusion of different types depending on the particular statistics of the trip-lengths or jumps. Nevertheless, the population spreads over all the space and the final state tends to have uniform density, which is not a very realistic feature in urban systems.

More elaborated theoretical frameworks add aspects such as continuous time movements to the random walks, so that agents can travel at any time and do not do it in a synchronised way, or fractional random walks in which the next jump is a product of a process with long memory with respect to the previous displacements. As occurs with Lévy flights, it is possible to consider ambivalent models in which both the space jumps and the waiting times follow power-laws. These ideas are motivated by empirical observations in different datasets [106, 111, 116, 117].

One important issue to mention is that individuals typically return to one or several locations, e.g. home or work. Taking as basis the previous random-walk-like frameworks, models have been proposed to include return to previous visited locations [117, 118]. Usually, the probability of returning is proportional to the number of visits that an individual has paid to a place, which defines the so-called preferential return [117]. The rest of the time the agents explore new environments. It has been also proposed that individuals’ interest in a place may also decline with time in some cases. This mechanism helps to modify the center of mass of each agent’s movements after a medium-long period of time and goes under the name of recency [119]. Other studies have shown that people visit regularly a finite number of places, and if a new place becomes frequent, another one is abandoned [120].

Humans are social animals, and, therefore, realistic models need to contemplate the effect of social interactions on mobility [121–123]. There are plenty of circumstances in which this becomes an important question: people may travel in family or in other generic groups; additionally, they may share a common destination synchronising their trajectories to meet somewhere. From a data point of view, traces of these behaviours can be observed using online social networks [124–126], cell phone records [127–129] and surveys [130, 131]. This phenomenon can help to improve individual model predictions on trajectories thanks to the correlations in displacements with the social contacts [132]. The effect of group mobility has been considered in transportation microsimulations since a little more than a decade ago [121–123, 133–138]. Interestingly, the relation between social networks and mobility is bidirectional. Our friends determine some of our mobility patterns, but we establish as well new social relations with people we meet in the visited locations. Traces of the organisation of mobility can be detected in the social network, which allowed for the definition of models characterising this interplay [139–141].

### Modelling aggregated mobility flows

Passing now to the aggregated models, generally trip flows are characterized as origin-destination (OD) matrices. Every element of the matrix at row *i* and column *j* conveys the information on the number of trips between areas *i* (origin) and *j* (destination). An OD matrix can also be represented as a directed weighted network, with links pointing from the area of origin to the one of destination and the weight standing for the number of trips. These matrices have been used traditionally to express the trip demand between zones of a city and are, therefore, an essential tool for infrastructure planning. Finding models able to infer the OD matrices from non-mobility variables is thus a question of great relevance. Traditionally, two family of approaches have dominated these endeavours: the intervening opportunities and the gravity models.

Intervening opportunities models were initially introduced in 1940 [142]. The idea guiding these models is that the population behaves as a source of trips and the destination depends on the number of opportunities an agent sees around her/his residential area. The probability of the agent to select one of these opportunities and, therefore, to set one destination for the agent’s trips relies on different functions that try to quantify the quality of the opportunity. These models have been profusely studied for a long time, see for example Refs. [143–148]. Recently, a self-consistent version called radiation model has been introduced [149]. The radiation model considers the “quality” of opportunities as a random variable and, as a consequence, the one to be selected should correspond to the largest quality value. The selection of extremes under these circumstances generates a few families of universal distributions, depending on the nature of the original random variable and its moments. This universality allows thus to close the expressions and find an equation for the flows of trips between areas. Later, other versions of the model have been considered to improve the treatment of the spatial scales and the nonlinear relation between opportunities quality and zone attractiveness [150–152].

The gravity model [153] takes the population of the origin as the source for the trips, so the trip number is proportional to it. The attractiveness of the destination area is related as well to its population, the relation can be linear in the simplest form of the model or, more generically, nonlinear. But the main question differentiating the gravity model from the intervening opportunities ones is that the flow of trips decays with the distance between origin and destination with a deterrence function. Most commonly, such a function can be an exponential or a power-law and its form may depend on the geographical scale considered, the purpose of the mobility or the transportation mode [154]. The simplicity of this law has made it very popular for applications, for instance, in transport infrastructure planning [155, 156], geography [146] and spatial economics [157, 158]. The gravity model can be deduced from a maximum entropy principle, as proposed by Sir Alan Wilson [159, 160], who is a pioneer in introducing the framework of statistical mechanics within geography, and more precisely within spatial interaction models [161], more than 50 years ago. It is also important to mention that the basic equation for the gravity model is unconstrained: given the population and distance between areas, one obtains directly an estimation of the flows. This is not the case for the radiation model, which is origin contained with the number of outgoing trips per area given as an input. Constraints can also be considered in gravity models, these can be at the origin, at the destination or at both [162, 163]. Assessing the effectiveness of these models is ongoing research [164], in some cases the scale of the system plays an important role [165], while in some others, the results from the radiation and gravity models are in agreement when assessing interventions [166].

### Comparison between models and future trends

In the case of commuting, there have been several works to compare the performance of both families of models. For example, the flows predicted by models with different levels of constraints are directly compared with the empirical values in Ref. [163]. The results seem to favour the exponential gravity model, even though by a narrow margin. More recently, a field theoretical framework for mobility has been proposed [167]. In this case, the average mobility of the flows out of each area are vectorially averaged and the different models have been used to explain the empirical patterns. Again, for the case of commuting the winner was a gravity model with exponential deterrence function, and in this case the margin with the other models was much wider.

Hierarchy in space also emerges naturally if one thinks about how the areas are embedded into one another: neighbourhoods form part of a city, cities lie within regions, regions constitute countries, etc. Recently, a model to exploit this hierarchical organisation has been proposed, where mobility at different scales gives rise to a nested structure of containers [101]. Finally, and recalling the Song’s model of preferential return [117], another model has been introduced exploring the role of the frequency of visits of individuals and mobility patterns, giving rise to a scaling law between the number of visitors, and the product of the visiting frequency and the travel distance [168].

## Conclusions

In the quest to model processes within cities, whole disciplines have emerged over the last century, from economics to transport modelling. Although each of these has produced great advancements, the time has come to couple them.

This brief overview, aimed at introducing the reader to several of the topics that are covered in this collection, as representative of the latest advancements in this field. These are, however, far from being comprehensive, and many important modelling and theoretical frameworks were left behind. For the individuals entering these topics, we aimed at provoking their curiosity by providing a brief view of the field and by pointing to some of the recent and open problems. For example, we initially discussed the need to include interactions and correlations within and between cities when accounting for agglomeration effects. Within the New Economic Geography, this is something that is well under consideration, and so the question of why within urban scaling laws these are widely overlooked, and only until very recently they are considered, is something that the researcher entering this field or developing further this field can no longer ignore. We also pointed out at the role of transport as an enabler of the interactions driving many of the observed processes. Although this has been recognised from very early on, many of the processes that are being considered when studying urban scaling laws, are not including it. Furthermore, many of the interactions take place in space, and we discussed how the morphology of a city has proven to play a substantial role in reinforcing certain patterns determining the location of functions. Overall, all these different layers, such as form and function, cannot be disconnected from the debate of agglomerations in cities. And it is the interplay between the different scales of interactions, enabled via different mechanisms, that give rise to the hierarchical organisation of urban systems.

Mobility was then discussed as an interrelated phenomenon with morphology, that encompasses the interactions between places. We introduced the two main modelling approaches to mobility: individual-based models, and models of aggregated mobility flows. These are the building blocks to entering such a prolific field that in the past decade has seen a surge driven by an increased availability of mobility data through mobile phones and other social media data, which has helped fine tune the models. Overall, agglomeration effects cannot be disconnected from the location of functions, nor from the differentiated opportunities given by transport, which are manifested through the mobility patterns left by people in a city. In this sense, the time is now ripe to try to integrate all these different components towards a better understanding of cities.
